# Influence of *Lactobacillus rhamnosus* Supplementation on the Glycaemic Index, Lipid Profile, and Microbiome of Healthy Elderly Subjects: A Preliminary Randomized Clinical Trial

**DOI:** 10.3390/foods13091293

**Published:** 2024-04-23

**Authors:** Chaiyavat Chaiyasut, Bhagavathi Sundaram Sivamaruthi, Subramanian Thangaleela, Natarajan Sisubalan, Muruganantham Bharathi, Suchanat Khongtan, Periyanaina Kesika, Sasithorn Sirilun, Thiwanya Choeisoongnern, Sartjin Peerajan, Pranom Fukngoen, Phakkharawat Sittiprapaporn, Wandee Rungseevijitprapa

**Affiliations:** 1 Innovation Center for Holistic Health, Nutraceuticals, and Cosmeceuticals, Faculty of Pharmacy, Chiang Mai University, Chiang Mai 50200, Thailand; chaiyavat@gmail.com (C.C.); sivamaruthi.b@cmu.ac.th (B.S.S.); sisubalan.n@cmu.ac.th (N.S.); kesika.p@cmu.ac.th (P.K.);; 2 Office of Research Administration, Chiang Mai University, Chiang Mai 50200, Thailand; 3 Department of Pharmaceutical Sciences, Faculty of Pharmacy, Chiang Mai University, Chiang Mai 50200, Thailand; 4 Neuropsychological Research Laboratory, Neuroscience Research Center, School of Anti-Aging and Regenerative Medicine, Mae Fah Luang University, Bangkok 10110, Thailand; 5 Health Innovation Institute, Chiang Mai 50200, Thailand; 6 Department of Pharmaceutical Chemistry and Technology, Faculty of Pharmaceutical Sciences, Ubon Ratchathani University, Ubon Ratchathani 34190, Thailand; 7 School of Cosmetic Science, Mae Fah Luang University, Muang District, Chiang Rai 57100, Thailand

**Keywords:** *Lactobacillus rhamnosus*, aging, gut microbiota, probiotics, lipid profile, microbiome

## Abstract

Aging is a time-dependent complex biological process of organisms with gradual deterioration of the anatomical and physiological functions. The role of gut microbiota is inevitable in the aging process. Probiotic interventions improve gut homeostasis and support healthy aging by enhancing beneficial species and microbial biodiversity in older adults. The present preliminary clinical trial delves into the impact of an 8-week *Lactobacillus rhamnosus* intervention (10 × 10^9^ CFU per day) on the glycaemic index, lipid profile, and microbiome of elderly subjects. Body weight, body fat, fasting blood glucose, total cholesterol, triglyceride, high-density lipoprotein, and low-density lipoprotein (LDL) are assessed at baseline (Week 0) and after treatment (Week 8) in placebo and probiotic groups. Gaussian regression analysis highlights a significant improvement in LDL cholesterol in the probiotic group (*p* = 0.045). Microbiome analysis reveals numeric changes in taxonomic abundance at various levels. At the phylum level, Proteobacteria increases its relative frequency (RF) from 14.79 ± 5.58 at baseline to 23.46 ± 8.02 at 8 weeks, though statistically insignificant (*p* = 0.100). Compared to the placebo group, probiotic supplementations significantly increased the proteobacteria abundance. Genus-level analysis indicates changes in the abundance of several microbes, including *Escherichia-Shigella*, *Akkermansia*, and *Bacteroides*, but only Butyricimonas showed a statistically significant level of reduction in its abundance. Probiotic supplementations significantly altered the *Escherichia-Shigella* and *Sutterella* abundance compared to the placebo group. At the species level, *Bacteroides vulgatus* substantially increases after probiotic treatment (*p* = 0.021). Alpha and beta diversity assessments depict subtle shifts in microbial composition. The study has limitations, including a small sample size, short study duration, single-strain probiotic use, and lack of long-term follow-up. Despite these constraints, the study provides valuable preliminary insights into the multifaceted impact of *L. rhamnosus* on elderly subjects. Further detailed studies are required to define the beneficial effect of *L. rhamnosus* on the health status of elderly subjects.

## 1. Introduction

Aging is cellular senescence, a complex biological process with progressive decline in anatomical and physiological functions of multiple cells and tissues. The irreversible aging process is genetically marked and regulated by genetic and environmental factors [1]. The accumulated cellular senescence effects during aging produce damage and disturb somatic maintenance, which could cause certain cellular and molecular distractions leading to genetic instability, epigenetic alterations, mitochondrial dysfunction, proteostasis loss, stem cell exhaustion, cellular senescence, deregulation of nutrient sensing, and so on [2]. The intestine has been recognized as a crucial organ because age-related pathologies are associated with gut microbiota imbalances and gut-associated immune systems. The gut microbiota forms a biological ecosystem with trillions of bacteria and fungi and maintains the host’s health and energy homeostasis. The composition of gut microbiota is relatively stable throughout adult life, designs the health conditions of the host by balancing the pathogens, nutritional status, and energy expenditure, and substantially changes with aging and results in age-associated diseases [3]. The microbial imbalance causes cellular senescence, wherein the cells undergo irreversible growth arrest and accumulate senescent cells during aging due to the development of a senescent-associated secretory phenotype. This comprises the secretion of pro-inflammatory cytokines, growth factors, chemokines, proteases, and extracellular matrix components [4]. The accumulated senescent cells in the skin cause dermatological disorders mediated by the gut–skin axis influenced by the gut microbiota and their metabolites [4]. The mutual relationship between the gut microbiota and the human host helps to protect against the colonization of pathogenic bacteria, metabolic activities, synthesizing bioactive peptide compounds, vitamins, and hormones, and managing the immune system [5,6].

The gut microbiota is stable throughout adulthood and fluctuates with aging and disease conditions [3]. The gut microbial composition changes in the elderly population compared to adults due to various factors such as the aging process, nutritional habits, lifestyle, stress, reduced intestinal functions [3,7], changes in immune responses, lack of physical activity, infections, use of medications, and hospitalization [8,9,10]. Generally, the gut microbiota in adults mostly comprises *Firmicutes* and *Bacteroides* and smaller populations of *Actinobacteria*, *Proteobacteria*, and *Verrucomicrobia* [11]. Aged peoples’ gut microbiota is abundant with Bacteroides and Proteobacteria with a decrease in *Firmicutes* and *Bifidobacteria* [9,12,13]. Elderly subjects have reduced bacterial diversity and beneficial bacteria. Also, the elderly subjects have lower *Bifidobacteria* and *Lactobacilli* levels and increased Enterobacteriaceae and *Clostridia* levels than young subjects [14,15]. Older adults showed a reduced proportion of butyrate-producing *Clostridium* cluster *XIVa*, *Roseburia* and *Ruminococcus* [16]. In long-living people, their intestinal microbiota abundantly presented with *Akkermansia*, *Bifidobacterium*, and *Christensenellaceae* [17,18]. In elderly subjects, the gut microbial composition changes, and the microbial diversity is reduced because of various factors such as the accumulation of pro-inflammatory microbes and reduced beneficial microbes [19], aging, nutritional and lifestyle changes, decreased gut functions, and stress [3]. The age-dependent changes in the gut microbiota mean the gradual changes in the microbial diversity involving the decline in the abundance of core dominant microbial species like *Bifidobacteria* counts and the ratio of *Firmicutes* to *Bacteroides* and the increase in the abundance of sub-dominant microbial species like *Proteobacteria* [17,20].

During aging, the changes in lipid metabolism and their metabolite levels cause an increase in body adiposity. Excess adiposity can cause lipid toxicity, leading to cardiovascular diseases, arthritis, cancer, type 2 diabetes, and Alzheimer’s disease [21]. In humans, with an increase in age, the blood glyceride levels tend to increase, and the white blood lysophosphatidylcholine levels decrease. Human longevity is associated with specific sphingolipid and phospholipid blood profile changes. Furthermore, certain lipids and lipid-related compounds have been found to change depending on age. Lipid-related interventions in various model organisms are capable of modulating the lifespan. It shows that lipid metabolism is linked to the aging process and may enhance the health span. Blood lipids and lipid-related molecules can be biomarkers for human aging studies [22]. Lactic acid bacteria (LAB) and bifidobacteria are found commonly in the gut of humans, with potential physiological benefits, such as enhancing gut function and increasing the uptake of micronutrients, lowering cholesterol, and protecting the gastrointestinal tract from infection by modulating immune functions [23]. It is well-known that an individual’s gut microbiota is shaped right from the gestational stage. Increasing evidence states that probiotic interventions and dietary patterns significantly influence or modulate age-associated changes in gut microbiota and immune functions, promoting healthy aging outcomes [24]. Although several clinical studies evaluated every aspect of aging consequences, anti-aging, or healthy aging properties, the effect of each probiotic strain widely varies on gut composition, immune function, and metabolite synthesis. Certain other studies investigated the involvement of probiotics in gut health, cognitive functions, lipid metabolism, and other biomarkers. In this study, we evaluated the impact of *Lactobacillus rhamnosus* interventions on glycaemic indexes, lipid profiles, and differences in the microbial diversity of healthy Thai elderly subjects.

## 2. Materials and Methods

The study protocols were approved by the Ethical Committee of Ubon Ratchathani University (Code: UBU-REC-44/2564). The participants’ consent was obtained before the study.

### 2.1. Study Group

**Figure 1:**
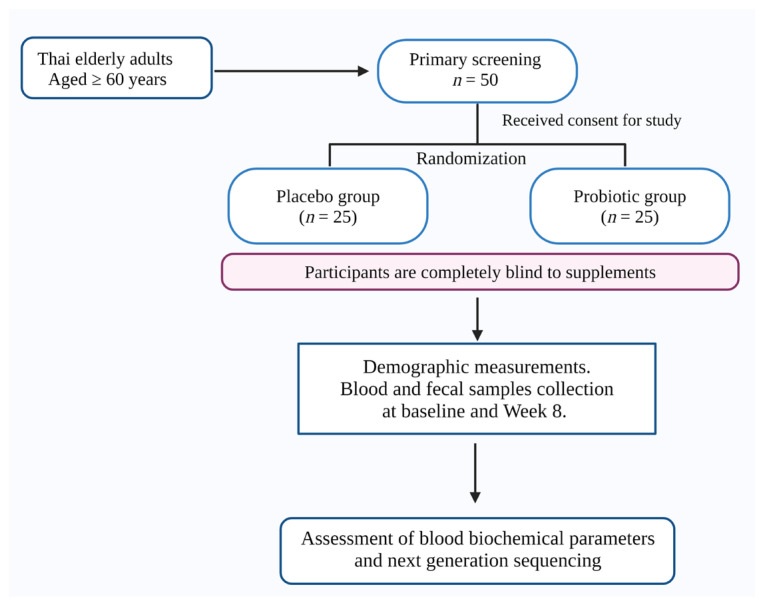
The illustration shows the study protocol (created using Biorender.com; accessed on 4 March 2024).

Thai elderly adults aged ≥ 60 who were willing to participate and complete the study were included. Participants under any other medications or taking probiotics in the previous 2 weeks were omitted from the study. After this primary screening, participants (*n* = 50; male =12; female = 38) were included in the study. The participants were randomly allotted into placebo (*n* = 25) and probiotic (*n* = 25) groups. Participants were completely blind to the supplements. The samples (blood and fecal) were gathered from the study subjects at baseline (week 0) and after 8 weeks. Participants were asked to follow the allocated follow-up visits without absence. The changes in the glycemic, lipid parameters and microbial composition were studied. The study protocol is illustrated in Figure 1.

### 2.2. Probiotics Supplementation

Aluminum foil sachets containing 10 × 10^9^ CFU of *Lactobacillus rhamnosus* received from Lactomason Co., Ltd. (Gyeongsangnam-do, Republic of Korea) were provided to the participants in the probiotic group. The placebo group participants were given a 10 g cornstarch sachet. All participants were instructed to take the supplement regularly by dissolving the contents of one sachet in a glass of water before breakfast. Instructions were given to store the sachet in a dry place at 2–8 °C. Participants were encouraged not to change their physical activities, nutrition, or lifestyle. The participants were advised to avoid the intake of any other probiotics, dietary supplements, or fermented food throughout the study.

### 2.3. Demographic Assessments

After assigning the participants to respective groups, the sociodemographic characteristics, including age, gender, smoking, alcohol drinking, and body weight, were recorded. Body and visceral fat were manually noted using an electronic scale (Picooc^®^, Model S1 Pro, Beijing, China) (Table 1). The blood parameters, including triglyceride, fasting blood glucose (FBS), total cholesterol (TC), high-density lipoprotein-cholesterol (HDL-C), and low-density lipoprotein-cholesterol (LDL-C), were determined from blood using the automated machine at AMS Clinical Service Center, Chiang Mai University, Chiang Mai, Thailand.

### 2.4. Next-Generation Sequencing (NGS)

The fecal genomic DNA was isolated, and DNA sequencing was conducted by the Omics Sciences and Bioinformatics Center, Faculty of Science, Chulalongkorn University, Thailand [25]. Due to the lack of quality DNA, we conducted NGS for only 12 samples each for the placebo and probiotic groups.

### 2.5. Statistical Analyses

The data are shown as the mean ± standard error of the mean (SEM) for continuous outcomes or as an absolute number and percentage for categorical outcomes. A paired *t*-test was performed for normally distributed data. The Wilcoxon signed-rank test analyzed the skewed data. The differentiation of outcomes between the placebo and probiotic groups was compared using a Mann–Whitney U test. The *p*-value < 0.05 was set as significant (two-tailed). The STATA version 15.1 for Windows (StataCorp, College Station, TX, USA) was utilized for statistical analysis.

## 3. Results

### 3.1. Changes in Biochemical Parameters

The basic parameters like body weight, body fat, visceral fat, and blood biochemical parameters like FBS, TC, TG, HDL, and LDL were measured at baseline and week 8 of the study. There were no significant differences in glycaemic or lipid parameters between the baseline and after treatment in the placebo group (Table 2). The marginal differences were observed in FBS, TG, HDL, and LDL after treatment in probiotic groups. These changes can be considered the initial state of functional changes due to the probiotic intervention. However, more duration is required to achieve significant effects in these parameters.

The statistical comparison of the considered parameters between the treatment and placebo groups was detailed (Table 3). The body weight (reduced; placebo (−0.06); probiotic (−0.68)), body fat (reduced; probiotic (−0.48)), visceral fat (reduced; probiotic (−1.45)), FBS (reduced; probiotic (−2.96)), and TC (reduced; probiotic (−14.36)), TG (reduced; probiotic (−7.88)), and LDL (reduced; probiotic (−5.44)) values were changed after the treatment in the placebo and probiotic groups, but not statistically significant (Table 3).

*L. rhamnosus* supplementation for 8 weeks substantially improved LDL (−69.85 to −0.79; *p* = 0.045) (Table 4). Besides LDL, no other parameters showed significant differences after 8 weeks of study.

### 3.2. Microbiome Analysis

The valid sequences were identified by matching the raw sequences with the corresponding barcode. The raw-sequence tags underwent analysis using QIIME 2™. Following chimera detection, the sequences were grouped into operational taxonomic units (OTUs) at a 97% sequence identity threshold. Quality assessment of pair-end reads was conducted by examining information in FASTQ files using DADA2. Reads failing to meet the default QIIME 2™ threshold values were filtered out, including a minimum quality score of 25, minimum/maximum length requirements of 200/1000, prohibition of ambiguous bases, and absence of mismatches in the primer sequence. The details of processed sequence reads are detailed in Table 5.

Changes in the phylum, genus, and species diversity between and within (baseline vs. after treatment) the placebo and probiotics group samples were compared. PW0 and PW8 indicate the baseline and after-treatment samples of the placebo group, respectively. TW0 and TW8 indicate the baseline and after-treatment samples of the probiotic group, respectively.

#### 3.2.1. Alpha Diversity

**Figure 2:**
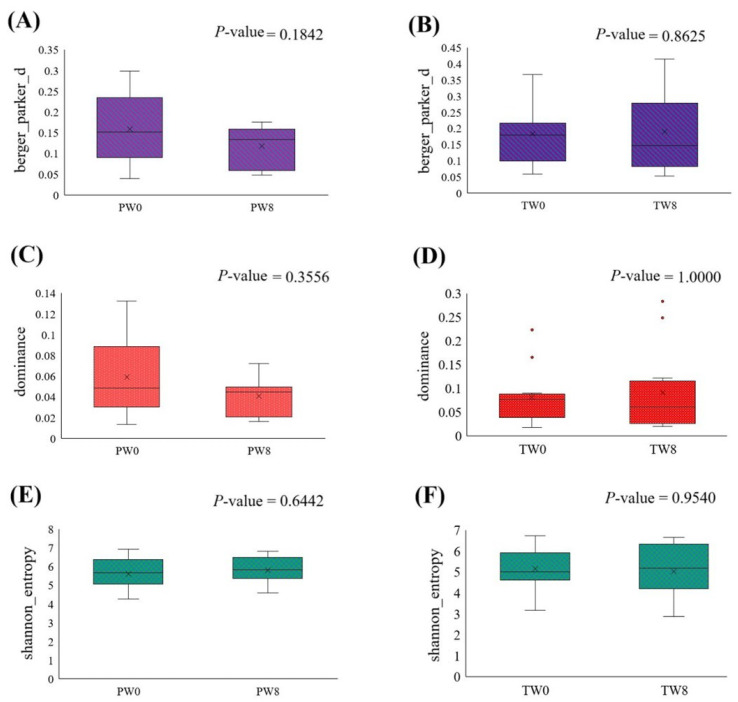
Alpha diversity estimation between the baseline and post-treatment of placebo (PW0 and PW8) and probiotic groups (TW0 and TW8). The results of Berger–Parker (**A**,**B**) analysis, dominance metrics (**C**,**D**), and Shannon entropy (**E**,**F**).

The Shannon diversity index was calculated to evaluate substantial group variations and analyzed using the Kruskal–Wallis (pairwise) test. Berger–Parker analysis was carried out to measure dominance within the community and the proportion of individuals belonging to the most abundant species in the sample. There were no significant changes observed in Berger–Parker analysis (Figure 2A,B), dominance metrics (Figure 2C,D), or Shannon entropy analysis (Figure 2E,F) after treatment in placebo and probiotic groups compared to the respective baseline. The results indicate that, despite observing numerical changes, there was no alteration in microbial abundance or consistency following the treatments.

#### 3.2.2. Beta Diversity

**Figure 3:**
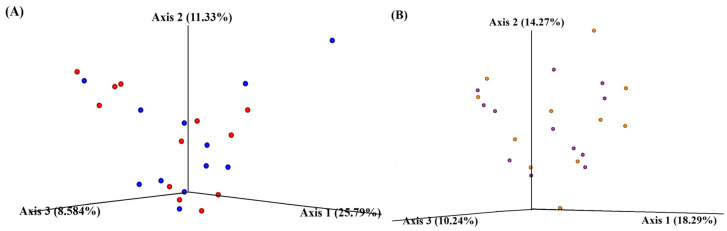
Beta diversity of placebo (**A**) and probiotic (**B**) group. The circle dots indicate the outlier samples. The red and blue dots indicate the baseline and treatment samples of the placebo group. The purple and orange dots indicate the baseline and treatment samples of the probiotic group.

Principal coordinate analysis (PCoA) was employed to assess the relationships between samples, with visualization facilitated by QIIME 2™ View. PCoA plots were generated based on the first three principal coordinates, annotated according to their variance. In the placebo group, 25.79, 11.33, and 8.58% microbial variations were observed in axes 1, 2, and 3, respectively. Similarly, microbial variations of 18.29, 14.27, and 10.24% were observed in axes 1, 2, and 3, respectively, in the probiotic group. The scattered dots, representing baseline and after-treatment samples, indicated that microbial diversity changed after treatments (Figure 3).

#### 3.2.3. Taxonomical Allocations and Determinations

**Figure 4:**
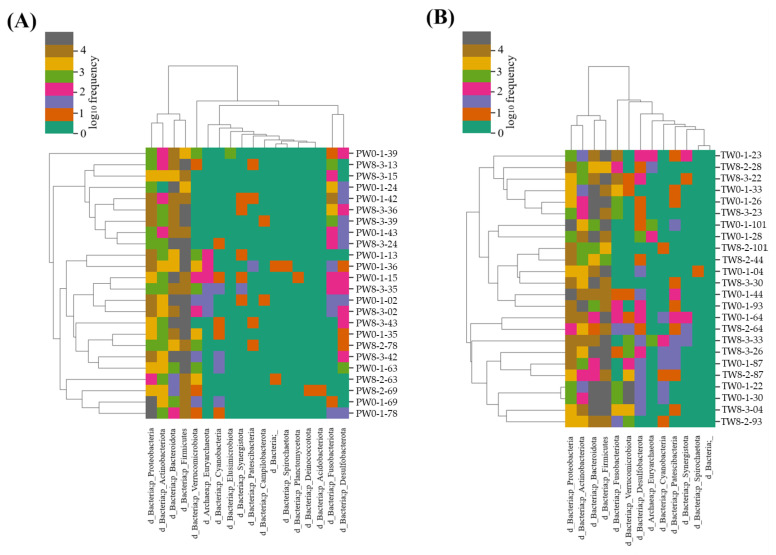
The heat-map represents the taxonomy estimated for the placebo (**A**) and probiotic (**B**) samples after week 0 and week 8 of treatment. The diversity was represented with log10 frequency.

A heat map was generated to illustrate the predicted bacterial taxonomy in the placebo and probiotic samples (Figure 4A,B).

The changes in microbial abundance after probiotic treatment are detailed in Table 6. There were numerical changes in the RF of the microbial abundance at the phylum level, but they were not statistically significant. Similarly, non-significant alteration of microbial abundance was observed at the genus and species level, except *Butyricimonas* (*p* = 0.017) and *Bacteroides vulgatus* (*p* = 0.021) (Table 6).

Table 7 details the changes in microbial abundance in the placebo group. The abundance of the phylum Actinobacteriota significantly (*p* = 0.023) increased after treatment. The abundance of *Escherichia-Shigella* (*p* = 0.050) was reduced, and the abundance of *Collinsella* (*p* = 0.045) and *Sutterella* (*p* = 0.047) increased significantly after treatment. No significant differences were reflected in species level in the placebo group (Table 7).

The comparison of placebo and probiotic groups after the treatment period indicated that proteobacteria (*p* = 0.015), *Escherichia-Shigella* (*p* = 0.024), *Sutterella* (*p* = 0.039), and *Bacteroides vulgatus* abundancy were altered significantly (Table 8).

## 4. Discussion

Age is a significant factor influencing the composition of the human intestinal microbiota, which changes throughout a person’s life [26,27,28,29]. These changes are influenced by factors such as physiological alterations in the gastrointestinal tract associated with aging, dietary habits specific to different countries, lifestyle choices, frailty conditions, antibiotic usage, and nutritional behaviors. Numerous studies have explored the impact of age-related physiological changes, lifestyle factors, and dietary habits on the gut microbiota [26,27,28,29,30]. Aging is accompanied by gut microbiome alterations that increase the various aging-associated diseases. It is difficult to conclude which factor initially contributed to this shift in the gut microbiome. However, lifestyle practices like increased medication, reduced mobility, and diet are found to manipulate the gut microbial composition. Understanding microbial manipulation in the elderly develops promising strategies to prevent age-associated diseases [31]. In the human body, gut microbiota acts as an important metabolic organ facilitating the metabolism of nutrients [32]. In addition to its association with metabolic diseases like obesity, liver diseases, intestinal diseases, neuropsychiatric diseases, diabetes, and cardiovascular diseases (CVD), the gut microbiota also acts as a reservoir for antibiotic-resistant genes [33]. The genotype of the host, diet, diseases, and age affect the composition and diversity of gut microbiota [34,35]. Therefore, employing microbial intervention to manipulate the gut microbiota represents an innovative strategy for impacting sleep and well-being [36]. Ingesting probiotics orally enables the restoration of functional activities within the gut microbiota [37].

Probiotic supplementation can protect gut integrity and restore its functions by initiating the growth of beneficial microbes, safeguarding the intestinal barrier, and positively modulating immune functions [1]. Costabile et al. investigated the effects of probiotic *L. rhamnosus GG* and pilus-deficient *L. rhamnosus GG-PB12*combined with soluble corn fiber on microbiota, immunity, metabolism, and blood lipids in healthy elder persons. Consumption of *L. rhamnosus* GG and prebiotics increased the natural killer cell activity compared to the baseline group. The fecal microbiota analysis showed that the synbiotic supplementation of *L. rhamnosus GG* with corn fiber and *L. rhamnosus GG-PB12* with corn fiber significantly increased *Parabacteroides*. *L. rhamnosus GG* with corn fiber increased *Ruminococcus*, and *Incertae Sedis* and decreased the *Oscillospira* and *Desulfovibrio. L. rhamnosus GG* and corn fiber further reduced the total cholesterol and LDL. *L. rhamnosus GG-PB12* with corn fiber treated volunteers showed a significant reduction in C-reactive protein compared to baseline. Thus, the dietary intervention with *L. rhamnosus* GG and corn fiber could positively enhance the immune response and microbial community [38]. The lipid status of an individual plays an important function in reducing the risk of CVD. Few observational studies suggested that hyperlipidemic subjects have a high risk of developing CVD. Additionally, it was found that reduced serum cholesterol, in turn, reduces the CVD risk. Clinical studies evaluated the beneficial effects of probiotic supplementation on serum lipid profiles [39,40,41]. Synbiotic interventions have been found to have more benefits in hypercholesterolemic patients than in normal people, and the reduction in total cholesterol and LDL levels is greater in the elderly than in younger individuals [38].

In this study, the difference between placebo and probiotic groups at the end of 8 weeks showed that the mean body weight reduction was almost the same. Reductions in visceral fat, FBS, TC, TG, and LDL were greater than placebo but not up to significant levels; simultaneously, there was a suggestive trend indicating improvement from baseline to week 8. However, after 8 weeks, this significance was reduced (*p* = 0.25) [36]. The difference in HDL level was increased 8 weeks after treatment (Table 3). Certain studies correlated that elevated serum TC, TG, and LDL and low HDL increase the risk for CVD [42,43,44]. Probiotics had no significant effects on the body, visceral fat, FBS, TC, TG, and HDL. The effect of probiotics on LDL depends on various factors, and a significant reduction in LDL was observed in the present study (Table 4). These results indicate that probiotic *L. rhamnosus* may improve the lipid profile when treated long or combined with other probiotics or prebiotic compounds.

In adults, Firmicutes are predominantly present in the gut, followed by Bacteroidetes [13]. Among the oldest adults, there is generally a decrease in Firmicutes and an increase in Bacteroidetes, which aligns with previous findings indicating a rise in the Firmicutes/Bacteroidetes ratio during adulthood followed by a decline in older age [45]. Vogt et al. [46] reported that *Bacteroidetes* showed an increase in abundance, whereas *Firmicutes* and the genus *Bifidobacterium* exhibited a decrease. Similarly, prior research has demonstrated that excessively high and excessively low Firmicutes/Bacteroidetes ratios can be linked to metabolic and gastrointestinal disorders [47]. These findings imply that a balanced distribution of these core phyla may signify good health and longevity, although specific environmental factors may partly influence this equilibrium. The abundance of Proteobacteria increased after *L. rhamnosus* supplementation; even though the changes were insignificant, it is noteworthy that *L. rhamnosus* supplementation could improve the microbiome positively in elderly subjects (Table 6). With the support of the evidence from the previous studies, proteobacteria, which is associated with increased gut inflammation and dysbiosis, was more abundant in older adults than in younger adults [48,49,50,51]. The Shannon–Wiener index considers fewer common species [37].

Following the treatment period, a comparison between the placebo and probiotic groups revealed significant alterations in the richness of *Escherichia-Shigella* (*p* = 0.024), *Sutterella* (*p* = 0.039), *Agathobacter* (*p* = 0.050), and *Bacteroides vulgatus* (*p* = 0.033) (Table 8). Laongkham et al. explained that the core gut microbiota of healthy Thai individuals comprises eleven species, including Firmicutes, Bacteroidetes, and Proteobacteria, shared by over 90% of subjects. Notably, *Escherichia coli* was found to be highly prevalent, especially among Thai elderly individuals. Age and PCA coordination were also correlated, particularly regarding the loading vector associated with the genus *Escherichia/Shigella*. However, no significant difference was observed in the abundance of this genus group between the adult and elderly groups [52]. After 8 weeks of probiotic intervention, non-significant changes were observed in the abundance of *Akkermansia* (*p* = 0.751), *Prevotella* (*p* = 0.609), and *Bifidobacterium* (*p* = 0.610) (Table 6). The alterations in *Akkermansia*, along with the changes in *Bifidobacterium* and the decline of *Prevotella*, have been proposed as biomarkers for PD [53].

In the case of the *Agathobacter* genus, López-García et al. mentioned that it did not meet statistical significance during the analysis. Still, there was a noticeable fluctuation in its occurrence throughout the clinical trial. In the *Lactiplantibacillus pentosus*-supplemented group, there was a rise in the average frequency of *Agathobacter* sequences from 4.67 to 4.98%. Conversely, there was a decrease in the placebo group from an initial frequency of 3.70% to a final frequency of 1.98% [54]. Similarly, in an investigation, nine healthy individuals were chosen to undergo a fasting regimen of approximately 17 h per day for 29 days. After the trial, no notable alterations in the measured values were reported. However, a noteworthy rise in the prevalence of both *Akkermansia muciniphila* and *Bacteroides fragilis* was reported [55].

Additionally, two clinical trials demonstrated a reduction in the abundance of *B. vulgatus* following the administration of probiotics [56,57]. Assessing intestinal microbiota composition poses a significant challenge due to its notable variability. This is primarily because modifications to commensal strains can occur within a short span of just a few days through changes in diet and lifestyle [58,59]. These findings underscore specific taxonomic changes within the probiotic group over the study period, emphasizing the importance of evaluating microbial dynamics at different taxonomic levels for a comprehensive understanding of the impact of probiotic intervention.

## 5. Limitations

The study has a few limitations that warrant consideration in interpreting its findings. The sample size of the study was relatively small, and the results may not be fully representative of the broader elderly population of Thailand. Individual variations among the study subjects may impact the generalizability of the observed effects. The study duration (8 weeks) was relatively short, with long-term effects. The sustainability of changes in glycaemic index, lipid profile, and microbiome may not be adequately captured within this timeframe.

Additionally, the exclusive use of a single strain, *L. rhamnosus*, as the probiotic intervention limits the understanding of potential synergistic effects that may arise from combining multiple strains. Furthermore, the study employed a single dosage of the probiotic, and exploring the impact of different dosage levels could provide insights into the dose–response relationship and optimal supplementation levels. The absence of follow-up research beyond the 8-week intervention period is another limitation, as longer-term assessments could reveal whether the observed effects persist or diminish over time.

## 6. Conclusions

The 8-week supplementation of *L. rhamnosus* improved the glycaemic index and lipid profile positively, but statistical significance was not observed. Gaussian regression analysis indicated that the probiotic supplementation significantly reduced the LDL level in the elderly subjects. Microbiome analysis revealed taxonomic shifts in both the placebo and probiotic groups. The results revealed that *L. rhamnosus* supplementation did not significantly affect the microbiome of the healthy elderly subjects. The study, however, is limited by its small sample size, short duration, and the use of a single probiotic strain. Future research with larger cohorts, extended study periods, and different probiotic formulations must confirm the findings. Addressing these limitations will contribute to a more comprehensive understanding of the potential benefits and mechanisms underlying probiotic interventions in the elderly population.

## Figures and Tables

**Table 1:** Sociodemographic data of the study participants.

No.	Variables	Group		*p*-Value
		Placebo (*n* = 25)	Probiotic (*n* = 25)	
1	Age	64.96 ± 0.86	63.00 ± 1.09	0.165 ^a^
2	Male, *n* (%)	3 (12.00)	9 (36.00)	0.095 ^b^
	Female, *n* (%)	22 (88.00)	16 (64.00)	
3	Smoking			0.490 ^b^
	No, *n* (%)	25 (100.00)	23 (92.00)	
	Yes, *n* (%)	0 (0.00)	2 (8.00)	
4	Alcohol drinking			0.235 ^b^
	No, *n* (%)	25 (100.00)	22 (88.00)	
	Yes, *n* (%)	0 (0.00)	3 (12.00)	
5	Body weight (kg)	63.80 ± 2.29	59.40 ± 2.17	0.170 ^a^
6	Body fat (%)	32.77 ± 1.41	27.76 ± 1.45	0.036 *^a^
7	Visceral fat (%)	15.18 ± 0.57	13.55 ± 1.49	0.060 ^c^
8	FBS (mg/dL)	106.12 ± 8.77	104.00 ± 5.97	0.843 ^a^
9	TC (mg/dL)	211.68 ± 9.41	209.12 ± 8.55	0.841 ^a^
10	TG (mg/dL)	134.48 ± 11.20	157.36 ± 12.49	0.179 ^a^
11	HDL (mg/dL)	53.04 ± 1.95	52.72 ± 3.09	0.931 ^a^
12	LDL (mg/dL)	131.08 ± 8.35	120.01 ± 6.22	0.293 ^a^

FBS: fasting blood sugar; TC: total cholesterol; TG: triglycerides; HDL: high-density lipoprotein; LDL: low-density lipoprotein. Data are mean ± SE. * indicates the significant difference in *p*-value at a 95% confidence interval. ^a^ *p*-value from the independent *t*-test, ^b^ *p*-value from Fischer’s exact test, ^c^ *p*-value from the Mann–Whitney U test.

**Table 2:** Biochemical parameters at baseline (week 0) and treatment (week 8) in the placebo and probiotic groups.

Parameters	Placebo (*n* = 25)		*p*-Value	Probiotic (*n* = 25)		*p*-Value
	Baseline (P-Week 0)	Treatment (P-Week 8)		Baseline (T-Week 0)	Treatment (T-Week 8)	
Body weight (kg)	63.80 ± 2.29	63.74 ± 2.30	0.884 ^a^	59.40 ± 2.17	58.73 ± 2.11	0.058 ^a^
Body fat (%)	32.77 ± 1.41	34.95 ± 1.53	0.127 ^a^	27.76 ± 1.45	27.28 ± 1.67	0.657 ^a^
Visceral fat (%)	15.18 ± 0.57	15.27 ± 0.66	0.402 ^b^	13.55 ± 1.49	12.09 ± 1.13	0.098 ^b^
FBS (mg/dL)	106.12 ± 8.77	109.92 ± 6.95	0.504 ^a^	104.00 ± 5.97	106.96 ± 5.84	0.375 ^a^
TC (mg/dL)	211.68 ± 9.41	201.96 ± 11.98	0.178 ^a^	209.12 ± 8.55	194.76 ± 7.63	0.055 ^a^
TG (mg/dL)	134.48 ± 11.20	149.80 ± 13.62	0.184 ^a^	157.36 ± 12.49	149.48 ± 12.62	0.463 ^a^
HDL (mg/dL)	53.04 ± 1.95	53.52 ± 3.55	0.858 ^a^	52.72 ± 3.09	56.28 ± 3.46	0.137 ^a^
LDL (mg/dL)	131.08 ± 8.35	128.42 ± 8.46	0.728 ^a^	120.01 ± 6.22	114.57 ± 8.00	0.439 ^a^

P-week 0: Placebo-week 0; P-week 8: Placebo-week 8; T-week 0: Treatment-week 0; T-week 8: Treatment-week 8; Baseline-Week 0; Treatment-Week 8; TC: total cholesterol; TG: triglycerides; HDL: high-density lipoprotein; LDL: low-density lipoprotein. Data are mean ± SE. ^a^ *p*-value from Paired *t*-test, ^b^ *p*-value from the Wilcoxon signed-rank test.

**Table 3:** The differentiation of biochemical parameters between the placebo and probiotic groups at the end of the study.

Variables	Difference		*p*-Value *
	Placebo (*n* = 25)	Probiotic (*n* = 25)	
Body weight (kg)	−0.06	−0.68	0.252
Body fat (%)	2.17	−0.48	0.163
Visceral fat (%)	0.09	−1.45	0.104
FBS (mg/dL)	3.80	−2.96	0.734
TC (mg/dL)	−9.72	−14.36	0.869
TG (mg/dL)	15.32	−7.88	0.479
HDL (mg/dL)	0.48	3.56	0.214
LDL (mg/dL)	−2.66	−5.44	0.823

FBS: Fasting blood sugar; TC: total cholesterol; TG: triglycerides; HDL: high-density lipoprotein; LDL: low-density lipoprotein. Data are mean. * *p*-value from the Mann–Whitney U test.

**Table 4:** Gaussian regression analysis of the probiotic treatment group after 8 weeks of study.

Parameters	Coefficient	95% Confidence Interval	*p*-Value
Body weight (kg)	−0.65	−2.24 to 0.94	0.409
Body fat (%)	−2.48	−6.48 to 1.52	0.214
Visceral fat (%)	−0.39	−2.14 to 1.36	0.650
FBS (mg/dL)	−5.19	−22.15 to 11.78	0.536
Total cholesterol (mg/dL)	−4.99	−28.91 to 18.93	0.675
Triglyceride (mg/dL)	−25.07	−74.60 to 24.47	0.308
HDL (mg/dL)	9.76	−2.43 to 21.94	0.112
LDL (mg/dL)	−35.32	−69.85 to −0.79	0.045 *

FBS: Fasting blood sugar; TC: total cholesterol; TG: triglycerides; HDL: high-density lipoprotein; LDL: low-density lipoprotein. Data are mean. * indicates the significant difference in *p*-value at a 95% confidence interval.

**Table 5:** The 16s rRNA amplicon sequences of the placebo (Baseline: PW0, After-treatment: PW8) and probiotics (Baseline: TW0, After-treatment: TW8) groups.

Sample-ID	Input	Filtered	Denoised	Merged	Non-Chimeric
Placebo group					
PW0-1-02	228,670	156,586	155,663	152,508	84,553
PW0-1-13	76,716	50,519	49,906	48,821	41,576
PW0-1-15	121,423	88,254	87,612	85,709	52,312
PW0-1-24	65,607	49,262	48,994	48,202	28,559
PW0-1-35	72,981	50,936	50,576	49,171	36,557
PW0-1-36	121,096	82,249	81,562	79,976	68,897
PW0-1-39	48,332	34,305	34,012	33,225	25,337
PW0-1-42	83,999	62,262	61,820	60,451	40,175
PW0-1-43	62,702	43,389	42,818	41,423	30,313
PW0-1-63	64,517	54,975	54,789	53,894	35,429
PW0-1-69	69,463	57,120	56,965	56,297	42,940
PW0-1-78	82,033	67,793	67,539	66,816	48,736
PW8-2-63	34,397	14,825	14,708	14,531	11,610
PW8-2-69	41,790	19,358	19,147	18,877	13,859
PW8-2-78	54,624	25,338	25,053	24,421	18,908
PW8-3-02	99,997	68,321	67,637	66,196	46,325
PW8-3-13	65,990	46,652	46,327	45,632	36,879
PW8-3-15	50,672	32,262	31,961	31,489	24,307
PW8-3-24	128,327	93,300	92,849	90,848	70,533
PW8-3-35	50,172	36,302	35,955	34,840	25,906
PW8-3-36	88,839	64,988	64,507	63,085	51,453
PW8-3-39	84,642	60,545	60,065	59,023	44,259
PW8-3-42	264,836	208,317	207,379	203,133	145,732
PW8-3-43	100,425	74,396	73,846	72,279	52,322
Probiotic group					
TW0-1-22	138,660	101,667	101,218	99,829	74,148
TW0-1-23	108,785	78,702	77,922	76,382	51,645
TW0-1-26	148,589	111,060	110,479	108,555	71,637
TW0-1-30	145,969	108,395	107,827	106,324	79,072
TW0-1-33	124,551	100,196	99,962	99,237	62,441
TW0-1-4	67,800	48,002	47,444	46,493	36,199
TW0-1-101	92,779	77,626	77,267	76,435	65,627
TW0-1-28	123,207	98,275	97,849	95,999	57,353
TW0-1-44	93,555	76,108	75,727	74,927	57,845
TW0-1-64	79,342	63,330	63,010	62,273	45,977
TW0-1-87	80,502	65,053	64,689	63,708	46,194
TW0-1-93	85,017	67,246	67,043	66,112	54,043
TW8-2-101	29,904	15,109	14,925	14,678	13,074
TW8-2-28	37,619	18,604	18,249	17,977	14,104
TW8-2-44	39,047	19,011	18,854	18,650	15,916
TW8-2-64	35,484	17,170	17,017	16,849	13,546
TW8-2-87	29,232	13,553	13,405	13,298	9193
TW8-2-93	51,548	25,060	24,826	24,404	19,697
TW8-3-22	80,475	57,923	57,475	56,476	49,362
TW8-3-23	88,815	66,880	66,592	65,661	50,791
TW8-3-26	240,804	181,521	180,569	177,129	145,464
TW8-3-30	87,286	64,381	64,027	63,254	50,862
TW8-3-33	137,037	94,961	94,181	92,399	81,152
TW8-3-4	128,895	93,093	92,460	90,949	76,221

**Table 6:** The statistical differences in the phylum, genus, and species between the baseline and after-treatment samples in the probiotic group.

Taxonomy	Baseline (TW0)	After Treatment (TW8)	*p*-Value
Phyla			
Proteobacteria	14.79 ± 5.58	23.46 ± 8.02	0.100
Verrucomicrobiota	5.54 ± 3.71	2.39 ± 1.84	0.475
Bacteroidota	27.77 ± 7.90	24.61 ± 6.31	0.938
Actinobacteriota	9.27 ± 4.00	6.66 ± 1.84	0.875
Firmicutes	41.09 ± 6.81	41.48 ± 6.57	0.754
Fusobacteriota	1.32 ± 0.80	1.27 ± 0.80	0.969
Desulfobacterota	0.19 ± 0.07	0.11 ± 0.05	0.388
Patescibacteria	0.04 ± 0.02	0.01 ± 0.004	0.254
Genera			
*Escherichia-Shigella*	13.71 ± 5.90	21.90 ± 8.63	0.117
*Akkermansia*	5.63 ± 3.77	2.49 ± 1.90	0.751
*Bacteroides*	16.81 ± 6.76	18.21 ± 4.90	0.272
*Bifidobacterium*	5.80 ± 3.86	4.30 ± 1.52	0.610
*Phascolarctobacterium*	4.66 ± 1.87	2.66 ± 1.15	0.724
*Prevotella*	9.58 ± 3.40	4.96 ± 2.78	0.609
*Faecalibacterium*	2.85 ± 0.74	5.75 ± 1.96	0.182
*Blautia*	5.52 ± 2.78	3.77 ± 2.17	0.239
*Collinsella*	1.70 ± 0.61	2.22 ± 0.77	0.388
*Weissella*	3.89 ± 3.70	0.07 ± 0.03	0.308
*Subdoligranulum*	0.79 ± 0.32	1.32 ± 0.48	0.638
*Agathobacter*	0.41 ± 0.13	1.13 ± 0.41	0.100
*Romboutsia*	0.52 ± 0.21	0.39 ± 0.14	1.000
*Roseburia*	0.72 ± 0.22	0.99 ± 0.55	0.784
*Alistipes*	0.45 ± 0.17	0.42 ± 0.36	0.325
*Paraprevotella*	2.05 ± 1.17	2.79 ± 2.40	0.305
*Streptococcus*	1.43 ± 0.90	1.38 ± 0.87	0.433
*Fusobacterium*	1.14 ± 0.85	0.38 ± 0.16	0.969
*Slackia*	0.84 ± 0.37	1.76 ± 1.12	0.906
*UCG 002*	0.69 ± 0.17	1.14 ± 0.28	0.410
*Dorea*	1.53 ± 1.24	1.09 ± 0.53	0.255
*Lactobacillus*	0.08 ± 0.02	0.39 ± 0.16	0.784
*Monoglobus*	0.05 ± 0.03	1.31 ± 1.18	0.365
*Parasutterella*	0.57 ± 0.43	0.55 ± 0.42	0.184
*Enterococcus*	0.22 ± 0.08	0.64 ± 0.33	0.723
*Butyricicoccus*	0.91 ± 0.74	0.08 ± 0.04	0.388
*Olsenella*	0.20 ± 0.11	0.72 ± 0.32	0.305
*CAG 352*	0.81 ± 0.39	0.30 ± 0.15	0.076
*Holdemanella*	0.33 ± 0.17	0.35 ± 0.11	0.145
*Fusicatenibacter*	0.45 ± 0.17	0.42 ± 0.36	0.583
*Parabacteroides*	0.66 ± 0.22	0.61 ± 0.21	0.969
*Coprococcus*	0.28 ± 0.14	0.69 ± 0.27	0.383
*Lachnoclostridium*	0.50 ± 0.15	0.30 ± 0.12	0.153
*Barnesiella*	0.38 ± 0.15	0.07 ± 0.04	0.178
*Odoribacter*	0.32 ± 0.10	0.17 ± 0.10	0.267
*Atopobium*	0.52 ± 0.51	0.06 ± 0.05	0.344
*Megamonas*	0.85 ± 0.35	0.39 ± 0.27	0.268
*Clostridia UCG 014*	0.09 ± 0.06	0.49 ± 0.37	0.767
*Klebsiella*	1.32 ± 0.63	0.48 ± 0.21	0.289
*Ruminococcus*	0.38 ± 0.24	0.24 ± 0.12	0.969
*Erysipelotrichaceae UCG 003*	0.31 ± 0.13	0.08 ± 0.04	0.234
*Enterobacteriaceae*	0.35 ± 0.18	0.19 ± 0.12	0.456
*Anaerostipes*	0.08 ± 0.03	0.38 ± 0.16	0.132
*Enterobacter*	0.21 ± 0.07	0.31 ± 0.20	0.505
*Veillonella*	0.37 ± 0.15	1.25 ± 1.14	0.326
*Flavonifractor*	0.10 ± 0.04	0.13 ± 0.10	1.000
*UBA1819*	0.09 ± 0.03	0.13 ± 0.07	0.875
*Lachnospiraceae UCG 010*	0.13 ± 0.09	0.04 ± 0.02	0.692
*Hungatella*	0.15 ± 0.12	0.15 ± 0.09	0.966
*Butyricimonas*	0.18 ± 0.05	0.06 ± 0.04	0.017 *
*Bilophila*	0.11 ± 0.06	0.11 ± 0.06	0.784
*Lachnospiraceae*	0.23 ± 0.06	0.26 ± 0.13	0.724
*Sutterella*	0.10 ± 0.04	0.15 ± 0.11	1.000
Species			
*Bacteroides stercoris*	10.31 ± 7.83	1.96 ± 1.01	0.422
*Bacteroides vulgatus*	12.34 ± 3.94	28.81 ± 8.31	0.021 *
*Bacteroides fragilis*	3.26 ± 1.61	13.96 ± 7.30	0.222
*Bacteroides uniformis*	10.70 ± 3.76	5.32 ± 2.28	0.365
*Lachnospiraceae NK4A136 group*	4.77 ± 2.02	6.29 ± 2.67	0.634
*Eubacterium hallii group*	12.40 ± 5.98	12.80 ± 5.46	0.610
*Ruminococcus torques group*	7.95 ± 2.23	6.66 ± 2.75	0.326
*Slackia isoflavoniconvertens*	7.76 ± 4.49	3.97 ± 1.77	0.579
*Bacteroides massiliensis*	6.64 ± 3.71	1.14 ± 0.62	0.148
*Eubacterium eligens group*	2.30 ± 0.96	1.12 ± 0.47	0.222
*Incertae sedis*	0.66 ± 0.22	0.56 ± 0.29	0.555
*Eubacterium ramulus*	0.48 ± 0.21	0.95 ± 0.42	0.969
*Parabacteroides distasonis*	1.49 ± 0.61	0.88 ± 0.49	0.422

The Wilcoxon signed-rank test was used to determine the statistical significance. * Statistically significant if *p* ≤ 0.05.

**Table 7:** The statistical differences in the phylum, genus, and species between the baseline and after-treatment samples in the placebo group.

Taxonomy	Baseline (PW0)	After Treatment (PW8)	*p*-Value
Phyla			
Proteobacteria	19.71 ± 6.45	7.66 ± 1.86	0.071
Bacteroidota	30.95 ± 8.51	24.05 ± 4.91	0.754
Firmicutes	44.75 ± 6.74	60.11 ± 2.45	0.136
Actinobacteriota	1.68 ± 0.46	4.35 ± 1.57	0.023 *
Fusobacteriota	1.69 ± 1.17	0.80 ± 0.50	0.938
Verrucomicrobiota	1.02 ± 0.39	2.85 ± 2.24	0.609
Desulfobacterota	0.21 ± 0.09	0.18 ± 0.06	0.969
Genera			
*Escherichia-Shigella*	16.30 ± 6.58	5.85 ± 2.14	0.050 *
*Bacteroides*	14.37 ± 4.14	14.92 ± 3.88	1.000
*Faecalibacterium*	3.22 ± 0.83	3.46 ± 1.24	0.530
*Blautia*	6.07 ± 3.24	5.11 ± 0.88	0.182
*Prevotella*	14.65 ± 6.52	6.97 ± 2.54	0.609
*Agathobacter*	1.90 ± 0.83	0.57 ± 0.24	0.224
*Collinsella*	0.98 ± 0.29	3.62 ± 1.89	0.045 *
*Subdoligranulum*	2.49 ± 1.04	1.20 ± 0.53	0.158
*Streptococcus*	2.13 ± 1.53	5.47 ± 3.56	0.695
*Roseburia*	1.33 ± 0.28	2.41 ± 0.83	0.195
*Phascolarctobacterium*	2.61 ± 0.55	3.78 ± 1.09	0.433
*Romboutsia*	0.46 ± 0.16	1.22 ± 0.51	0.081
*Klebsiella*	1.54 ± 0.59	0.63 ± 0.22	0.170
*Fusobacterium*	1.75 ± 1.21	0.92 ± 0.57	0.938
*Ruminococcus*	0.92 ± 0.33	1.11 ± 0.46	0.906
*Dorea*	0.64 ± 0.17	1.20 ± 0.53	0.254
*Akkermansia*	1.12 ± 0.43	3.92 ± 3.04	0.609
*Lachnoclostridium*	0.77 ± 0.14	1.28 ± 0.49	0.814
*UCG-002*	1.50 ± 0.55	0.95 ± 0.44	0.289
*Fusicatenibacter*	0.68 ± 0.28	0.35 ± 0.25	0.209
*Enterobacter*	1.29 ± 0.60	0.09 ± 0.03	0.090
*Veillonella*	0.21 ± 0.08	4.91 ± 2.59	0.100
*Alistipes*	1.23 ± 0.54	0.96 ± 0.38	0.480
*CAG-352*	0.39 ± 0.12	0.52 ± 0.22	0.969
*Enterobacteriaceae*	1.88 ± 1.10	0.35 ± 0.17	0.209
*Coprococcus*	0.70 ± 0.23	0.77 ± 0.22	0.410
*Sutterella*	0.49 ± 0.24	1.64 ± 0.99	0.047 *
*Anaerostipes*	0.41 ± 0.18	0.64 ± 0.22	0.556
*Parabacteroides*	0.90 ± 0.29	1.04 ± 0.35	0.875
*Holdemanella*	0.68 ± 0.37	0.53 ± 0.32	0.906
*Butyricicoccus*	0.52 ± 0.14	1.03 ± 0.25	0.100
*Enterococcus*	0.15 ± 0.09	2.62 ± 1.83	0.057
*Lachnospira*	0.41 ± 0.20	0.22 ± 0.10	0.428
*Lachnospiraceae*	0.62 ± 0.24	1.04 ± 0.31	0.255
*Paraprevotella*	0.58 ± 0.39	0.42 ± 0.25	0.969
*Muribaculaceae*	0.38 ± 0.18	0.95 ± 0.54	0.937
*Haemophilus*	0.10 ± 0.05	0.26 ± 0.15	0.178
*Bifidobacterium*	0.13 ± 0.05	0.50 ± 0.22	0.170
*UBA1819*	0.06 ± 0.02	0.10 ± 0.05	0.969
*Lachnospiraceae UCG010*	0.16 ± 0.04	0.14 ± 0.05	0.692
*Odoribacter*	0.15 ± 0.06	0.17 ± 0.10	0.937
*Oscillibacter*	0.05 ± 0.01	0.13 ± 0.08	0.937
*Bilophila*	0.13 ± 0.10	0.07 ± 0.03	0.783
*Butyricimonas*	0.26 ± 0.12	0.15 ± 0.06	0.844
Species			
*Bacteroides uniformis*	7.72 ± 2.21	5.48 ± 3.86	0.158
*Bacteroides vulgatus*	7.99 ± 2.23	8.17 ± 2.28	0.875
*Bacteroides plebeius*	5.44 ± 3.84	4.16 ± 2.49	0.937
*Lachnospiraceae NK4A136 group*	2.39 ± 1.72	3.78 ± 1.52	0.665
*Bacteroides coprophilus*	3.10 ± 2.96	2.03 ± 1.32	1.000
*Eubacterium hallii group*	11.47 ± 6.49	9.85 ± 5.06	0.754
*Ruminococcus gnavus group*	0.84 ± 0.38	7.56 ± 4.52	0.812
*Ruminococcus bicirculans*	1.81 ± 0.82	1.70 ± 0.80	0.906
*Lactobacillus salivarius*	7.42 ± 7.29	4.51 ± 4.36	0.902
*Prevotella stercorea*	3.32 ± 1.81	2.87 ± 1.50	0.475
*Prevotella copri*	1.55 ± 1.36	10.04 ± 4.89	0.475
*Ruminococcus torques group*	2.35 ± 0.48	3.51 ± 1.05	0.194
*Bacteroides stercoris*	4.60 ± 1.84	1.11 ± 0.59	0.194
*Parabacteroides merdae*	1.30 ± 0.43	0.90 ± 0.30	0.255
*Eubacterium eligens group*	1.28 ± 0.36	1.36 ± 0.63	0.433
*Parabacteroides distasonis*	2.05 ± 1.14	1.41 ± 0.81	0.692
*Ruminococcus gauvreauii group*	0.71 ± 0.36	1.75 ± 1.45	0.410
*Alistipes shahii*	0.87 ± 0.45	0.46 ± 0.23	0.194

The Wilcoxon signed-rank test was used to determine the statistical significance. * Statistically significant if *p* ≤ 0.05.

**Table 8:** The differentiation of microbiome between the placebo and probiotic groups at the end of the study.

Taxonomy	Placebo vs. Treatment		*p*-Value
Phylum			
Proteobacteria	−12.06	8.67	0.015 *
Bacteroidota	−6.90	−3.15	0.773
Firmicutes	15.36	0.39	0.166
Actinobacteriota	2.67	−2.61	0.564
Fusobacteriota	−0.89	−0.05	0.817
Verrucomicrobiota	1.84	−3.15	0.384
Desulfobacterota	−0.03	−0.07	0.686
Genus			
*Escherichia-Shigella*	−10.45	8.20	0.024 *
*Bacteroides*	0.55	1.40	0.326
*Faecalibacterium*	0.24	2.90	0.273
*Blautia*	−0.96	−1.76	0.106
*Prevotella*	−7.68	−4.61	0.908
*Agathobacter*	−1.33	0.72	0.050
*Collinsella*	2.63	0.52	0.525
*Subdoligranulum*	−1.29	0.53	0.133
*Streptococcus*	3.34	0.75	0.225
*Roseburia*	1.08	0.27	0.237
*Phascolarctobacterium*	1.18	−1.99	0.419
*Romboutsia*	0.76	−0.12	0.138
*Klebsiella*	−0.91	−0.83	0.977
*Fusobacterium*	−0.83	−0.05	0.817
*Ruminococcus*	0.19	−0.14	0.862
*Dorea*	0.55	0.45	0.817
*Akkermansia*	2.80	−3.14	0.487
*Lachnoclostridium*	0.50	−0.20	0.453
*UCG-002*	−0.55	0.92	0.260
*Fusicatenibacter*	−0.33	0.02	0.204
*Enterobacter*	−1.19	0.10	0.355
*Veillonella*	4.70	0.88	0.106
*Alistipes*	−0.28	−0.25	1.000
*CAG-352*	0.13	0.52	0.148
*Enterobacteriaceae*	−1.53	−0.16	0.419
*Coprococcus*	0.07	0.40	0.977
*Sutterella*	1.15	0.05	0.039 *
*Anaerostipes*	0.23	0.30	0.470
*Parabacteroides*	0.13	−0.04	0.908
*Holdemanella*	−0.16	−0.50	0.386
*Butyricicoccus*	0.51	0.43	0.326
*Enterococcus*	2.47	−0.02	0.182
*Lachnospiraceae*	0.41	0.03	0.312
*Paraprevotella*	−0.16	−0.03	0.541
*Bifidobacterium*	0.37	−1.50	0.795
*UBA1819*	0.04	0.04	0.729
*Lachnospiraceae UCG-010*	−0.02	−0.09	0.931
*Odoribacter*	0.03	−0.15	0.309
*Bilophila*	−0.06	0.00	0.862
*Butyricimonas*	−0.11	−0.12	0.271
Species			
*Bacteroides uniformis*	−2.24	−5.38	0.908
*Bacteroides vulgatus*	0.18	16.47	0.033 *
*Lachnospiraceae NK4A136 group*	1.39	1.52	1.000
*Bacteroides stercoris*	−3.49	−8.35	0.727
*Parabacteroides distasonis*	−0.64	−0.61	0.447

* Statistically significant if *p* ≤ 0.05.

## Data Availability

The original contributions presented in the study are included in the article, further inquiries can be directed to the corresponding author.
